# Professor Ferrier's New Scottish Philosophy
*Scottish Philosophy—The Old and the New. A Statement by Professor Ferrier.


**Published:** 1857-01-01

**Authors:** 


### Art. II.—PROFESSOR FERRIER'S NEW SCOTTISH PHILOSOPHY.*

Why Professor Ferrier should give to his system of philosophy a title which was only gradually extended as a mark of honour to the one it proposes to supersede,—and this, too, when it is not an extension, but a subversion, of the older system,—we leave to those who love to study character. To have won for his speculations a right to the title with which he has dignified them, he should at least have built upon the old foundation; and even then the imposition of so proud a name he should have left for others.

Reid won his laurels in defending the citadel of truth against the assaults of idealism and scepticism; and how did he do this? By stoutly contending for the veracity of the primary deliverances of the mind. Philosophers had given undue prominence to demonstration as the source of philosophic truth. To those fundamental beliefs which afford the data from which we reason, they had assigned a very subordinate place. Seeking to prove all things, they, one after the other, wandered into the maze of scepticism, or fell into the dream of idealism. But proof must at last repose upon self-evident truths. Here, then, is a source of truth independent of proof: on it Reid took his stand. True, it was that on which all, except philosophers, already took their stand. So much the better. Indeed, its universal recognition as a foundation evinced that it was the right one. Reid therefore decided upon siding with the "vulgar" against philosophers. "We are necessitated by reasoning," says Hume, "to contradict

* Scottish Philosophy—The Old and the New. A Statement by Professor Ferrier.

the primary instincts of nature, and to embrace a new system with regard to the evidences of our senses." "It is a bold philosophy," says Reid, "that rejects without ceremony principles which irresistibly govern the belief and the conduct of all mankind in the common concerns of life; and to which the philosopher himself must yield, after he imagines he hath confuted them. Such principles are older and of more authority than philosophy: she rests upon them as her basis, not they upon her."

But had Reid done no more than vindicate the superiority of our primary convictions over every derivative assurance, he would have accomplished little more than most unsophisticated scholars do when they encounter the deformed half-truths of idealism, and the offensive insinuations of scepticism. Besides thus standing forth as the champion of common sense against the philosophy of the period—in which he was not original, being only the mouthpiece of the unphilosophizing millions—he laboured hard to quell the feud that had existed so long between common sense and reasoning, with regard to the cardinal point of philosophy; and it was his success in this important undertaking that constituted his originality as a mental philosopher. Hence, in a letter to Dr. Gregory he writes:—"It would be want of candour not to own that I think there is some merit in what you are pleased to call *my philosophy;* but I think it lies chiefly in having called in question the common theory of *ideas, or images of things in the mind,* being the only objects of thought—a theory founded on natural prejudices, and so universally received as to be interwoven with the structure of language."

Herein, then, consists the spirit of the Scotch philosophy; it maintains the veracity of those primary convictions which all men have in common, as to the existence, for example, of the material world as a *non-ego.* Reid, finding it impossible to doubt such convictions, determined to accept no theory of outward cognition that did not confirm them. The received theory was at variance with our fundamental beliefs: this was enough to satisfy Reid that it was erroneous. He consequently, with all the humility and patience of the true inductive spirit, resolved to find out the true explanation of his spontaneous assurances; and if he did not reach the desired land, he obtained a view of it, and left for his successors the result of his valuable labours.

Sir William Hamilton, with the strong spontaneous assurances of his predecessor, with prodigious powers of memory, yet with reflective talent second to none, performed for the common-sense philosophy what its most sanguine admirers could have wished. He corrected some of its errors; he laid its foundation deeper by a more rigid—we do not say complete—analysis, and brought to its aid, from the prodigious store of his learning, the corroborative statements of philosophy from the dawn of speculation to the present day.

Now, however much Sir Wm. Hamilton has had occasion to differ from his predecessor, Reid; and however much he has reduced that philosopher's first truths to ulterior principles, he has always remained faithful to the spontaneous convictions of mankind; he is a true promoter of the genuine Scottish philosophy. He contends that every one who aspires to the name of philosopher should stanchly maintain, in opposition to any negative doctrine falsifying our fundamental beliefs, what is universally, because necessarily, believed; and "that nature is not gratuitously to be assumed to work, not only in vain, but in counteraction of herself; our faculty of knowledge is not, without a ground, to be supposed an instrument of illusion; man, unless the melancholy fact be proved, is not to be held organized for the attainment, and actuated by the love of truth, only to become the dupe and victim of a perfidious Creator."

It is not necessary, then, to admit the first truths of Reid as final; but it is all-important that we hold them to be truths, even though we have not yet arrived at the ground of their certainty. To call them vulgar prejudices that will not stand the scrutiny of philosophy, is grievously to mistake their nature; at all events, they are not on a par with ordinary prejudices; they are universal, and not to be dispelled, either by effort or by argument. The belief that the sun turned round the earth, did not exclude the contrary belief, as the issue proves; but the conviction that the mere material world exists, does, at least practically, exclude the contrary conviction; and this being the case, he is the truest philosopher who, admitting the authority of our spontaneous assurances, seeks the ground of it, rather than he who seems to rejoice in proving it to be fallible, as if what is universally believed must of course be philosophically false and vulgar.

Now, Professor Ferrier, although he claims for his system the paternity of Scotland, certainly cannot call it an extension of the older system; for being opposed to the primary cognitions of mankind, it must be similarly opposed to the conceptions of Reid, Stewart, and Hamilton; he consequently errs in giving his system a name that has so long been associated with a system radically different. "I hold," says Professor Ferrier, "that philosophy exists for the sole purpose of correcting the natural inadvertences of loose, ordinary thinking—that is her true and proper vocation; the old school, on the contrary, are of opinion that philosophy exists for the very purpose of ratifying, and if possible systematizing, these inadvertences. This is held by Reid and his followers to be the proper business of metaphysical science."

The proper business of Reid, Stewart, and Hamilton, has been to ratify and attempt to systematize the inadvertences of loose ordinary thinking! What a waste of time and talent! But who calls the convictions that Reid and his followers attempted to ratify, inadvertences? Professor Ferrier. What poor deluded mortals we are. But then, if we sit at the feet of Professor Ferrier, what shall we profit by it? we shall still have to carry our delusions with us to the grave. But are we, in the words of the truly great philosopher we have recently lost, really and truly "the dupes of a perfidious Creator." Monstrous supposition! No; what Professor Ferrier brands, with a veritable Coriolanus air, as the natural inadvertences of loose ordinary thinking, we shall continue to call spontaneous truths—truths that result from the constitution of the human mind, and which admit of being speculatively established; but which certainly can never be upset by any apparent demonstration—which every demonstration must be that leads us to regard them as inadvertences. Doubtless, Professor Ferrier is led away by the stubbornness of his reasoning; but where valid reasoning leads to such conclusions (O ye of little faith), there must be some flaw in the premises. Men of his cast of thought prefer admitting a demonstration to distrusting a pet principle which they have been at enormous pains to excogitate. Men like Reid and Hamilton prefer doubting the principle, to admitting an inference at variance with their primary convictions.

Without entering into the details of Professor Ferrier's system, let us simply examine his statement—that the *mere* material world has no real and absolute existence. His argument is as follows:—"The only material world which truly exists is one which either actually is, or may possibly be known. But the only material world which either actually is, or may possibly be known, is one along with which intelligence is, and must be, also known. Therefore the material world which truly exists is one, along with which intelligence also exists. Therefore the *mere* material world has no real and absolute existence."

We propose amending this argument by the addition of two small words: it will then run thus:—The only material world which truly exists (*for us*), &c. But the only material world, &c. Therefore the only material world which truly exists (*for us*) is one along with which intelligence also exists. To the argument thus worded there certainly can be raised no just objection. But then it does not warrant the sweeping conclusion, that the *mere* material world has no real and absolute existence: this would be to prove more than is contained in the premises.

Now if there is really no *mere* material world, our only authority for the statement is consciousness. But how can consciousness, from the knowledge it has of the *non-ego*, infer that the latter exists solely in connexion with intelligence? Only in one way: consciousness must be proved inseparably necessary to the absolute existence of the material world; so that if consciousness were to cease, the *non-ego* would cease as well; but to be known being a passive condition imposed upon the object by the active process of knowing, we have *primâ facie* evidence that it must exist antecedently to the imposition of this condition upon it by consciousness; for this condition is only a constituent of its *known* existence, not of its unknown. Let us enter into the matter more fully.

An object can only exist *for us* in connexion with intelligence. True: yet consciousness declares the object to be a *non-ego, and if consciousness is veracious*, a *non-ego* it is: then it is a *non-ego-cognised*: yet the *non-ego* is only causally, paternally* related to consciousness—that is, it is a *genus* paternally related to its *differentia*-cognised, forming the *species—non-ego-cognised.* Now, to this species, or synthesis, either element is indispensable; take away the *non-ego*, you destroy it; take away *cognised*, you destroy it likewise—you destroy the synthesis in which alone the *non-ego* exists *for us*. Therefore, *for us* there exists no mere material world, no *non-ego minus cognised.*

But, on the other hand, the *genus*, *non-ego*, being only paternally related to the *differentia*-cognised, the latter admits, in reality, of being separated from its *genus*, without obliging us to conceive that the *genus* is destroyed by the separation. "Admits, in reality, of being separated from its *genus*." What do you mean by that? In an act of perception, there are two elements, forming what we shall call, with your permission, a biune fact (object, *plus* consciousness); the minimum of existence *for us* being that contained in this biunity—that is, existence with this condition imposed upon it. Now, we mean that from this biune fact the object, element, is constantly passing away, leaving the other element as the remainder to record its presentation; but when it passes away, what becomes of it? Are we forced to infer that the separation of the other element from it involves its non-existence? By no means. Consciousness does not enter into its composition as a constituent part: it is a *non-consciousness*, consequently we cannot infer that, apart from consciousness, it does *not* exist; but can we prove that, apart from consciousness, it *does* exist? We believe we can—our argument is as follows:

In the order of knowledge, which is first to us, objects suppose consciousness (no consciousness no objects for us); but in the

* We postulate this proposition here, referring the reader to the sequel for the proof.

order of existence consciousness supposes objects (no objects no consciousness). Now, since consciousness in the order of existence supposes objects, the latter must be chronologically prior to the former, and consequently in the perception are only paternally related to consciousness in the order of existence, while in the order of knowledge the reverse is the case—that is, consciousness is causally related to objects in so far as they are *known* (not in so far as they *exist*) ; and it is the exclusive contemplation of this subjective side of the truth that leads to the incredible conclusions of idealism. Let us regard, as we ought, the subjective and objective sides of the truth with equal veneration, and then we have the philosophy of our spontaneous assurances—a philosophy not exposing their perfidiousness, but establishing their veracity.

There is a marked tendency among many philosophers to admit nothing into the category of existence but facts of consciousness; meaning the *fact* of its testimony, in contradistinction to the *truth* of the same. They admit the existence of the traveller, but deny his narrative, or deem it unworthy of credit. Now, in so doing, they are unconsciously undermining the ground they stand upon. Consciousness reports its own existence—reports all its changes—so then, at the foundation of all existence, we have a communication, or revelation. This is *the ultimate fact*, even that on the credibility of which we admit the existence of consciousness of the revealing power itself. Now, if consciousness be not allowed to speak decisively concerning what, by its own showing, is *not* itself; neither can it be allowed to speak decisively concerning itself;* "for the maxim '*falsus in uno, falsus in omnibus*,' must determine the credibility of consciousness, as the credibility of every other witness." It matters not how conclusive your proof may be that consciousness exists,—and here we shall quote the clearest we know of,—namely, "that in doubting the fact of his consciousness, the sceptic must at least affirm the fact of his doubt; but to affirm a doubt is to affirm the *consciousness* of it; the doubt would, therefore, be self-contradictory,—*i. e.* annihilate itself." It matters not, we repeat, how conclusive this proof may be, if the deliverances of consciousness are in the least incredible ; for this proof supposes them,† and therefore cannot be more credible than they are. So then we have no alternative left us, but to concede to consciousness the power of knowing a real *non-ego*. We can perfectly enter into the words of Stewart, therefore, when he says, " that the belief which accompanies consciousness, as to the present existence of its appropriate phenomena, rests on no foun-

* Hamilton's edition of Reid's works, page 746.

† As concerns *us*, everything that exists supposes the veracity of consciousness.

dation more solid than our belief of the existence of external objects;" and we cannot fully coincide with Sir William Hamilton's views on this point.

Stewart means, of course, that *practically* we have as good ground for believing in the existence of external objects, as in that of internal phenomena; and that this should be sufficient to win our *speculative* belief in the existence of either. Hamilton thinks this a mistake. If it is a mistake, it must be one in the speculative, not the practical sense. Stewart may not be justified in stating that philosophers had no better reason for admitting the existence of consciousness than of external objects, because Descartes, according to Hamilton and Cousin, had by a reasoning established the existence of the thinking subject, whereas no one had by a reasoning established the existence of external objects. Sir William Hamilton, relying on the conclusiveness of the said reasoning, gives up the argument from common sense, in respect to the *fact* of the testimony of consciousness; and only considers it in so far as it enables us to vindicate the *truth* of the testimony of consciousness. While Reid and Stewart then assert that common sense is our ultimate authority, subjectively as well as objectively, Hamilton recognises "a reasoning" as our ultimate ground of certitude in the subjective sphere; while in the objective sphere, he thinks with Reid that implicit faith in the deliverances of consciousness is amply justified. We regard Sir William Hamilton's contributions to philosophy, then, as in a state of transition from pure common-sense views to pure speculative. He gives what he considers a philosophical confirmation of the existence of consciousness, and its phenomena; in fact, he apparently establishes, in a speculative sense, the first half of the doctrine of common sense. But why did he stop here, and lead us to suppose that speculative reasoning had no more to conquer.

Having gained possession of the subjective sphere, will it content itself with anything less than the objective as well? We think not; and had Sir William Hamilton boldly pursued the path he followed thus far, he would have arrived at a full speculative confirmation of the faith of Stewart, which he now considers a mistake. We feel assured that the mistake is on Hamilton's side, and that Stewart's faith is more to be relied on than his critic's inference. We feel convinced that consciousness *is* no more credible, and that speculative reason can discover no *better* ground for admitting its credibility, when it asserts its own existence, than when it asserts the existence of the *non-ego*. The veracity of consciousness is the ultimate fact, which we can neither *prove*, nor *disprove*, without committing a *petitio principii*; and we cannot admit the testimony of consciousness as a

*fact*, without supposing the *veracity* of consciousness in revealing such a fact; and if we distrust our sensible perceptions, we are compelled also to doubt the very existence of the same, for, if they are not competent witnesses in their declarations concerning external objects, neither are they competent to report their own existence: for, *as concerns us, everything that exists supposes the veracity of consciousness.*

This leads us to remark that Mr. Ferrier, like many others, has been led astray by want of implicit faith in the integrity of consciousness. Did he believe that this involved the reception of the first truths of Reid as final? That he could not do, when many of them had already been reduced, or were in process of being reduced, to simpler elements. "The first truths of the old Scottish school have not only no value in philosophy, they have no value in any intellectual market in the world," says the Professor. Perhaps so, yet they are legitimate results of laws of thought, laws which at first have only an *implicit* manifestation, and depend upon reflection for their *explicit* development.

Perhaps the statement, that consciousness is trustworthy when its demands are complied with, is of no value in philosophy; yet certainly the proof of the statement must be a philosophical acquisition of the very first value; and, were it established, Professor Ferrier would have to concede that the *what* of existence, even in a philosophical sense, must be *simply* apprehended, before anything pertaining to it can be logically apprehended.

To come to the point, Mr. Ferrier states, that after much elaborate demonstration, and in opposition to the whole teaching of psychology, he has *proved* that existence is a compound, and not a simple; in technical language, that it is a synthesis of subject and object—a union of mind, and something else—which is not so strictly mind as mind itself is mind.* Now, in opposition to this, we state that consciousness has an immediate apprehension, an intuitive cognition of an object as a *non-ego*, i.e. *not* a union of mind and something else. This is a simple and ultimate deliverance of consciousness, a deliverance universally

* The writer in Blackwood's, June, 1842, on Berkeley and Idealism, has these words:—"Nature herself, we may say, has so *beaten up together* sight and colour, that man's faculty of abstraction is utterly powerless to dissolve the charmed union. The two (supposed) elements are not two, but only one; for they cannot be separated in thought, even by the craft of the subtlest analysis. It is God's synthesis, and man cannot analyse it." Professor Ferrier says—"The *mere* material world has no real and absolute existence. But neither is it a nonentity (I am no Idealist); for there is no nonentity, any more than there is entity out of relation to all intelligence." The writer in Blackwood's also says—"It is perfectly true that the existence of matter depends entirely on the presence—that is, either the real or the ideal presence—of a conscious mind. But it does not follow from this that there would be *no matter* if no such conscious mind were present, or thought of as present; because *no matter* depends just as much upon the real or the ideal presence of a conscious mind." What are we to infer from these coincidences?

acted upon. But some men are not disposed to confide in it speculatively, unless some reason can be discovered to exclude the supposition of its being untrustworthy. Must we, therefore, prove the existence of external objects, or cease to contend for their reality? By no means: we have two alternatives still, which must be destroyed before we are brought to such a pass as that. In the first place, if *we* cannot prove the *existence* of the *mere* material world, *you* cannot prove its *non-existence*. So far then it is a drawn battle, but now we have a reserve to bring up, and you have not. We have an immediate perception of an object *as* numerically different from the conscious subject. It is more rational, therefore, to believe in the reality of the external world than not. But, in the second place, if we cannot prove the independent existence of the *non-ego*, it still remains competent for us to admit its existence without direct proof, and seek only to *prove* the veracity of consciousness in general, and consequently in its immediate perceptions. Well, then, is consciousness possessed of infallible integrity? We refer to what we have written above; but, let us ask, what part of the mind is it which demands to be thus satisfied? Certainly not the primary faculties. Immediate perception assuredly does not doubt its own sincerity. The conviction that there is an object numerically distinct from our apprehension of it is the very essence of sensible perception; the absence of such a conviction involves the absence of the perception also. Whence, then, the suspicion as to its truthfulness? It is one of reasoning's raising; it is entirely speculative; it cannot be practically entertained, *i.e.*, having the perception we cannot by any amount of effort will away the conviction which is absolutely essential to its existence. Now, reasoning can be satisfied with nothing less than absolute demonstration. Is it possible to render it the satisfaction it demands? Sir William Hamilton has done very much to make speculative doubt of no reputation, but he has not done enough to exclude it for ever from every sane mind. He has certainly *apparently* shown that in themselves as apprehended facts, or actual manifestations, the deliverances of consciousness are above all scepticism; but what we think of this, we have already stated. We think that Hamilton, since he evinced a disposition to grant the claims of reasoning in the subjective sphere, should have gone farther, or, at least, have admitted the possibility of reasoning's ratifying *all* the deliverances of common sense, whether they regarded self or not self.

We cannot avoid thinking that the Scotch philosophy has not exactly realized its intention. We think that while contending for a source of truth prior to demonstration, in which it is quite right, it carries the contest too far, and usurps the province of reasoning. For instance, Reasoning asks to be assured of the faithfulness of immediate perception, and Common Sense puts on the air of wounded honour, and feels surprised how Reasoning can be so basely suspicious, indeed so insane, as to doubt for a moment the integrity of a friend—a friend in whom she must practically confide, whether she will or not. But poor Reasoning is really wronged by such usage ; she has a right to make her demand, and will continue to make it till all doubt disappears.

Let us fully understand the point. Reasoning not only demands proof of the veracity of the primary deliverances of consciousness, but even calls itself to account, and seeks to prove that it is not self-deceived. Now, we humbly conceive that the Scotch school, as well as defending Common Sense against Reasoning, should have defended Reasoning against itself (*i.e.*, reasoning regarding its objects, against reasoning regarding itself). The Scotch philosophy, we conceive, when rightly understood, is a protest against the practice of openly or virtually doubting the veracity of the mind—of our reasoning as well as of our intuitive faculties ; and it errs when it takes upon itself to interdict any inquiry that speculative reasoning, which is not satisfied with the mere testimony of immediate perception, may make for its own peculiar gratification. But we must hasten back to the road from which we have digressed.

We have shown that many truths have to be received on the testimony of intuitive perception alone, and that the only proof they are susceptible of is an indirect one, namely, a proof of the veracity of consciousness. Had Prof. Ferrier sought to demonstrate this veracity, he would have done wisely ; but attempting, as he does, to prove what is immediately apprehended, is simply an abuse of one's reasoning powers.

We stated above, that consciousness is veracious when its demands are complied with. The first clause of this sentence we have just considered ; it remains for us to say something also about the second. The demands of consciousness we call, in other words, *its forms*. Now, neglecting the question of veracity, and striving to discover the form in which we discover new truths or principles, we call to mind Bacon. Neglecting to discover the form in which consciousness acquires first principles, but adopting the ancient deductive method, and labouring to establish the existence of consciousness as a basis of certitude, we call to mind Descartes. Bacon's method has been productive of an immense accumulation of scientific truth. Can we say the same of Descartes' ? We cannot ; and why ? Because he missed the forms, without the observance of which the principle of certitude does not apply ; whereas, if the forms be observed, the principle applies, though it be yet unnoticed.

Having shown that consciousness was the ground of all certitude, had Descartes substituted as the condition of that certitude Bacon's rules instead of the axiom—All clear ideas are true, what very different fruit would his method have borne. Consciousness is not to be regarded as a voucher for truth, when ideas are merely clear and distinct, as the result of the Cartesian philosophy so deplorably evinces. Indeed, Leibnitz's improvement upon this last, although a step in advance, does not quite bridge over the chasm which divides the inductive from the deductive method. And Mr. Ferrier, evidently continuing the same search, has yet to learn that the principle of contradiction is an essential part of inductive reasoning, and has not found in his pages its true place and formal enouncement. We mean to say, that if truths were not previously felt to be necessary, Prof. Ferrier's test would not prove them to be such. Take an example: Two straight lines cannot enclose space; you question the universality of this truth, how are you to be convinced? By laying down the counter-statement, Two straight lines can enclose space; we then perceive that this contradicts the conception which we must form of two straight lines. But must it do so for ever. Must it do so in the moon? The test is only effectual, as far as we can apply it in any number of conceptions we may choose to call up. But any number of such testings does not amount to infinitude, and consequently does not necessitate a universal conclusion. If then, for the sake of argument, we do not admit the proposition, that two straight lines cannot enclose space, to be universally true, we do not perceive that Mr. Ferrier's criterium forces us to do so; because it is not proved to us that in the counter-statement the predicate must necessarily, must always, be subversive of its subject. Enclosing space must be proved to be the positive cause of the absence of straightness in the two lines; and not enclosing space the negative cause of the presence of straightness in them, before we are warranted to conclude *formally* that two straight lines can *never* enclose space. But how is this to be done? That is the question. Prof. Ferrier must go considerably farther into the interior yet.

While upon this subject, we would take the opportunity of stating that we have evidence of two kinds of necessity; and that we think that Professor Ferrier comes down rather hard upon Mr. Cairns for suggesting, after Sir William Hamilton, that there is more than one. There is—1st. The necessary junction of one fact with another, perceived by us as an inference necessitated by the comparison of two propositions; and, 2nd. There is the universal junction of one fact with another, wherever or whenever that other exists, perceived by us as an inference necessitated by the law of contradiction; and now we would intimate to Mr. Ferrier that a counter-statement is not necessarily contradictory to a proposition, the necessity of which is not previously proved, or capable of being proved, as alluded to above. (No. 1.)

Before we conclude, we cannot help recording our conviction that Professor Ferrier has done well in so forcibly drawing attention to the not sufficiently recognised fact, that, *as concerns us*, objects only exist in the biune fact of perception ; but then, like most system-builders, he makes his truth four-square, when it is only half that. He errs in supposing that the subject-object is an essential part of the object-object ; and he is right in asserting that the object-object only exists *for us* in connexion with the subject-object; but again he errs in supposing that the external world can exist only under the condition in which it exists *for us*. Perception, so far as we have been able to analyse its contents, is—I. Consciousness. 1st, revealing itself; 2nd, as revealing a not-self—*plus*. II. The not-self, which it reveals. Revealing itself, as revealing a not-self, is the one element, the not-self is the other; and they are numerically different—the knowledge element, for instance, is not a component part of the other element. Professor Ferrier, therefore, commits an immense oversight in not severing consciousness as a *self*-object from the *not*-self object, when consciousness itself does this in *so positive a manner*. "Positively truly," says Mr. Ferrier, "but inadvertently!" This is the natural result of placing the *fact* of the testimony of consciousness before the *truth* of that testimony—the derived before the ultimate.

---

